# Analysis of plant microbe interactions in the era of next generation sequencing technologies

**DOI:** 10.3389/fpls.2014.00216

**Published:** 2014-05-21

**Authors:** Claudia Knief

**Affiliations:** Institute of Crop Science and Resource Conservation—Molecular Biology of the Rhizosphere, Faculty of Agriculture, University of BonnBonn, Germany

**Keywords:** next generation sequencing, plant microbiota, phyllosphere, rhizosphere, metagenomics, amplicon sequencing

## Abstract

Next generation sequencing (NGS) technologies have impressively accelerated research in biological science during the last years by enabling the production of large volumes of sequence data to a drastically lower price per base, compared to traditional sequencing methods. The recent and ongoing developments in the field allow addressing research questions in plant-microbe biology that were not conceivable just a few years ago. The present review provides an overview of NGS technologies and their usefulness for the analysis of microorganisms that live in association with plants. Possible limitations of the different sequencing systems, in particular sources of errors and bias, are critically discussed and methods are disclosed that help to overcome these shortcomings. A focus will be on the application of NGS methods in metagenomic studies, including the analysis of microbial communities by amplicon sequencing, which can be considered as a targeted metagenomic approach. Different applications of NGS technologies are exemplified by selected research articles that address the biology of the plant associated microbiota to demonstrate the worth of the new methods.

## Introduction

Plants live in association with diverse microorganisms, which thrive below ground in the rhizosphere and above in the phyllosphere (Vorholt, 2012; Bulgarelli et al., 2013). They are found as endophytes within the plant, as epiphytes attached on plant surfaces and in the nearby soil around the roots. These microorganisms can have beneficial, neutral, or detrimental effects on plant health and development (Newton et al., 2010). The majority of the diverse plant colonizing microorganisms follows a commensal lifestyle; they do not cause obvious harm to the plant, nor do they exert a strong plant growth promoting effect as known for instance from symbiotic nitrogen-fixing bacteria or mycorrhizal fungi. The opening questions to better understand the association between plants and their associated microbiota are the “Who is there?” and “What are they doing?” These are extended by “How do they life under given conditions?” “How do they respond to environmental changes and perturbations?” “How do they interact with each other?” and “How do they affect plant health and development?” Finding answers to these questions will lead to a better understanding of the association between microorganisms and plants; a prerequisite to assess if and how associated microorganisms may be used in the future to support plant growth and improve crop yield.

DNA based studies of the plant associated microbiota are of high value to address the aforementioned questions. Genomic analyses of individual microbial strains or metagenomic studies of whole microbial communities provide insight into the composition and physiological potential of plant associated microorganisms. RNA based studies can extend such studies in order to elucidate the actual metabolic activities and regulatory mechanisms of the microbial cells under given conditions. NGS technologies have a tremendous impact on DNA and RNA based analysis methods; they allow finding answers to questions that could not be addressed before, largely due to technical and financial limitations. Thus, plant microbe associations can now be studied at a speed and depth as never before.

The present review summarizes the main features of the currently available NGS systems and gives a brief outlook about what may be expected in the future. It critically discusses limitations of NGS platforms and shows up ways to compensate these. Applications in the context of plant-microbe-interactions are highlighted that profit from these new technologies, focusing on metagenomic analyses.

## Next generation sequencing technologies

Different NGS systems have in common that they produce a massive amount of sequencing data (up to gigabases and soon even terabases) in parallel. Often, NGS instruments are classified as second and third generation sequencing technologies (e.g., Schadt et al., 2010; Niedringhaus et al., 2011; Pareek et al., 2011; Liu et al., 2012). There is no consistent definition for this terminology, and it is difficult to assign all different instruments unambiguously to one or the other category (Schadt et al., 2010; Thompson and Milos, 2011). In this review I refer to all those methods that depend on a PCR step for signal intensification prior to sequencing as second generation sequencing instruments, opposed to single molecule sequencing. Second generation sequencing technology includes the 454 instruments from Roche, the different Illumina platforms and the Life Technologies instruments, i.e., the Sequencing by Oligonucleotide Ligation and Detection (SOLiD) and Ion Torrent sequencers. The only third generation sequencing instrument that is currently commercially available is the PacBio RS by Pacific Biosciences.

## Common and distinct features of second generation sequencing technologies

**Figure 1:**
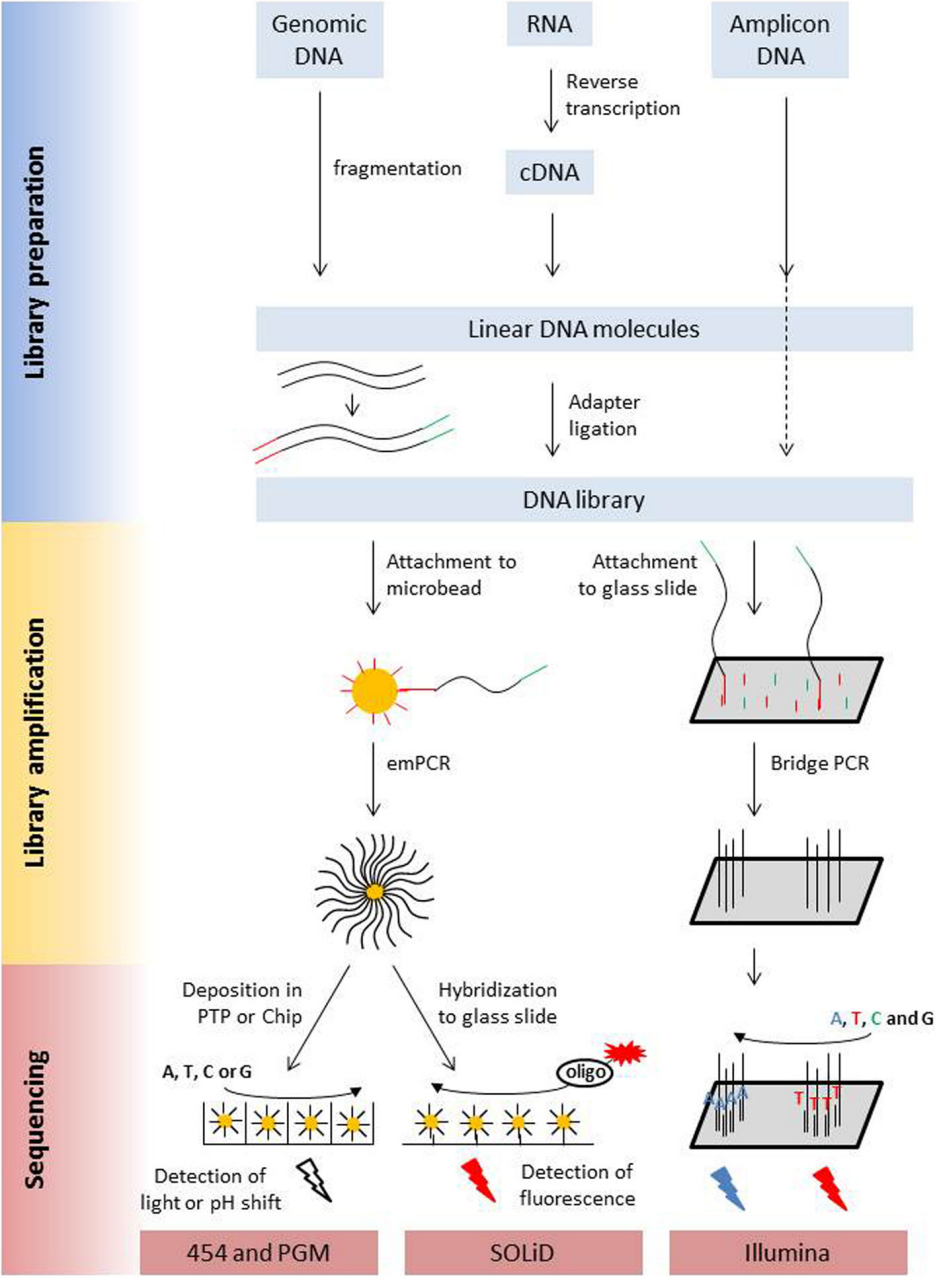
**Schematic presentation of the library preparation and sequencing process of the most commonly used next generation sequencing platforms**. All different types of starting molecules are converted into doublestranded DNA molecules that are flanked by adapters. Adapters are sequencing platform specific and enable the binding of the library molecules to surfaces, either beads or a flow cell, where they are amplified prior to sequencing. Clonal amplicons are spatially separated on the glass slides, chips, or picotiterplate. Sequencing is either a sequencing by ligation process with fluorescently labeled oligonucleotides of known sequence (SOLiD) or a sequencing by synthesis process. During Illumina sequencing, four differently labeled nucleotides are flushed over the flow cell in multiple cycles, depending on the desired read length. During 454 and Ion PGM sequencing unlabeled nucleotides are flushed in a sequential order over the flow cell. Incorporation is detected via a coupled light reaction (454) or the detection of proton release during nucleotide incorporation.

The main characteristics of NGS sequencers are described here in a comparative way in order to point out similarities and differences. A detailed description of second generation sequencing platforms and principles can be found in dedicated reviews (e.g., Voelkerding et al., 2009; Metzker, 2010; Glenn, 2011; Pareek et al., 2011; Zhang et al., 2011; Liu et al., 2012; Shokralla et al., 2012; Mardis, 2013; Morey et al., 2013). Despite differences in terms of sequencing principle, all current second generation sequencing platforms have several shared features with regard to library preparation, library amplification and the sequencing process (Figure 1, Table 1).

**Table 1:** **Technological specifications of currently commercially available next generation sequencing platforms**.

**Company (former companies)**	**Platforms**	**Library amplification**	**Carrier of library molecules or beads during sequencing**	**Sequencing principle**	**Nucleotide modifications**	**Signal detection method**	**Dominant type of sequencing error**
Roche (454 until 2006)	454 FLX Titanium	emPCR on microbeads	Picotiterplate	Pyrosequencing	None (except for dATP, which is added as thiol derivative dATPαS)	Optical detection of light, emitted in secondary reactions initiated by release of PP_i_ upon nucleotide incorporation	Indels in homopolymeric regions
	454 FLX+						
	454 GS Junior Titanium						
Illumina (Solexa until 2007)	Illumina GAIIx	Bridge-PCR on flow cell surface	Flow cell	Reversible terminator sequencing by synthesis	End-blocked fluorescent nucleotides	Optical detection of fluorescent emission from incorporated dye-labeled nucleotides	Substitutions, in particular at the end of the read
	Illumina HiSeq1000						
	Illumina HiSeq1500						
	Illumina HiSeq2000						
	Illumina HiSeq2500						
	Illumina MiSeq						
	Illumina NextSeq 500						
	Illumina HiSeq X ten						
Life Technologies (Agencourt until 2006, Applied Biosystems until 2008)	SOLiD 4	emPCR on microbeads; PCR on FlowChip surface for the 5500 W models	FlowChip	Sequencing by ligation	2-base encoded fluorescent oligonucleotides	Optical detection of fluorescent emission from ligated dye-labeled oligonucleotides	Substitutions, in particular at the end of the read
	SOLiD 5500						
	SOLiD 5500xl						
	SOLiD 5500 W						
	SOLiD 5500xl W						
Life Technologies (Ion Torrent until 2010)	Ion PGM	emPCR on microbeads	Ion Chip, a semiconductor chip	Semiconductor-based sequencing by synthesis	None	Transistor-based detection of H^+^ shift upon nucleotide incorporation	Indels
	Ion Proton						
Pacific biosciences	PacBio RS	Not applied	SMRT cell	Single-molecule, real-time DNA sequencing by synthesis	Phosphor-linked fluorescent nucleotides	Real-time optical detection of fluorescent dye in polymerase active site during incorporation	Indels

### Library preparation

Library preparation can be done from DNA (genomic or PCR amplified fragments) or RNA as input material. The latter has to be converted into cDNA during the library preparation process, direct sequencing of RNA is not yet possible. Due to size limitations for library molecules, genomic DNA and often also mRNA is fragmented, which is usually done mechanically, e.g., by sonication or nebulization, or enzymatically. The fragment size of a library is critical and depends on the sequencing platform that is going to be used. The standard fragment size of Illumina libraries is between 300 and 550 bp including adapters. Longer fragments up to 800 bp can be sequenced if cluster density on the flow cell is reduced to prevent interference of library molecules during the sequencing process. The size of libraries prepared for 454 sequencing depends on the sequencing run conditions. To obtain long reads with a modal length of 700 bp, a size of approximately 1500 bp is recommended. Libraries prepared for sequencing on the small-scale 454 Junior instrument or for sequencing using the older FLX chemistry should be smaller (300–750 bp). Libraries that are sequenced on the Ion Torrent Personal Genome Machine (PGM) platform should never be longer than the requested read length.

Libraries are constructed by adding sequencing platform-specific DNA adapters to the DNA molecules. This enables binding of the library fragments to a surface, which is either a microbead (454, Ion PGM, SOLiD) or a glass slide (Illumina, SOLiD). Moreover, the adapters allow amplification of the library fragments by emulsion PCR (emPCR) or bridge PCR. When amplicons are sequenced, e.g., in microbial community analyses, adapters are often already added during PCR using fusion primer constructs.

Diverse library preparation kits are commercially available and even more protocols have been published that are adapted to the specific needs of research projects. During the last years, library preparation methods were streamlined to reduce costs and preparation time and to enable high throughput library preparation on automated systems (e.g., Adey et al., 2010; Caruccio, 2011; Neiman et al., 2012; Rohland and Reich, 2012; Langevin et al., 2013) Methods were also optimized to reduce potential bias, e.g., by excluding PCR amplification steps (Kozarewa et al., 2009; Adey et al., 2010; Mamanova and Turner, 2011; Oyola et al., 2012; Van Dijk et al., 2014). Another goal is the reduction of the amount of input material. This ranges from several micrograms down to hundreds of pictograms (e.g., Adey et al., 2010; Tariq et al., 2011; Parkinson et al., 2012; Bowman et al., 2013; Langevin et al., 2013). In microbial metagenomic studies, which often aim at in-depth analysis of gene diversity, it is advisable to prepare libraries from microgram amounts of input material to cover as much of the diversity as possible and obtain high sequencing depth. It also has to be considered that library preparation from just a few nanograms of input material will require additional PCR steps to amplify the material, which is a potential source of bias.

Library construction using standard methods can easily be outsourced. If library preparation is done by oneself, care has to be taken that the generated libraries are compatible with the sequencing platform that is used for sequencing, as adapters were in some cases modified since the release of the first instruments. For instance, the sequencing of libraries that are constructed according to an Illumina GAIIx protocol is not necessarily fully supported on HiSeq or MiSeq instruments. Details should be discussed prior to the preparation of libraries with the sequence provider.

### Barcoding of libraries

At least one of the library adapters usually carries a library specific DNA sequence, often a 6- to 12-mer, referred to as barcode, molecular identifier (MID) or tag. This barcode enables the pooling of different libraries, which can then be further processed and sequenced within the same region of a picotiterplate (454), a lane of a flow cell (Illumina, SOLiD) or on a chip (Ion PGM). Barcoding allows sequencing of a complex set of libraries at rather low depth, which is of particular interest in large-scale ecological or biodiversity studies comprising many samples. In amplicon sequencing projects, a sample specific barcode is often already added during PCR amplification of the target genes to enable parallel sample processing at an early step. It should be noted that bias may be introduced when using complex fusion primers with adapters and different barcodes. This can be compensated to certain extent by using a two-step PCR procedure (Berry et al., 2011).

Several different barcode sets have been developed by hand or using software tools. They vary in length and account more and more strictly for different types of sequencing errors and sequencing platform specific needs to maximize data output (Faircloth and Glenn, 2012 and references therein; Kircher et al., 2012; Buschmann and Bystrykh, 2013; Costea et al., 2013). In some articles the use of a dual barcoding strategy is proposed for paired end sequencing in order to decrease sample misidentification rate or to decrease the number of individually tagged PCR primers (Gloor et al., 2010; Carlsen et al., 2012; Degnan and Ochman, 2012; Kircher et al., 2012; Kozich et al., 2013).

### Library amplification by emulsion PCR or bridge PCR

PCR amplification of the library molecules is required to increase signal intensity for the sequencing process. Amplification has to occur spatially separated for the individual library fragments on microbeads (454, PGM, SOLiD) via emPCR or on a glass surface (Illumina, SOLiD) via bridge PCR. Hybridization of the library fragments to the surfaces occurs via the adapters to surface-bound oligonucleotides. In the bead based method, each bead obtains only a single library molecule. The beads are spatially separated from each other during emPCR in individual water droplets in a water-oil emulsion. Beads with successfully amplified fragments are enriched and deposited in a picotiterplate (454), a semiconductor chip (Ion PGM) or hybridized to a glass surface (SOLiD) for sequencing. When library molecules are directly hybridized to a glass surface, their density on the surface has to be sufficiently low to prevent interference of library molecules, even after fragment amplification via bridge PCR (Figure 1).

Since the production and recovery of successfully templated beads from the water-oil emulsion during emPCR is time consuming, technically challenging and rather expensive, sequencing companies search for alternative methods to amplify library molecules. This has been realized in the recently released Wildfire technology for the SOLiD sequencer (SOLiD 5500 W) and is under development for Ion Torrent sequencers (Merriman et al., 2012).

### The sequencing process

Sequencing is performed in a massively parallel manner for ten thousands to billions of library fragments. It occurs via repeated cycles of nucleotide addition by a DNA polymerase or ligase (SOLiD), detection of incorporated nucleotides and washing steps. Due to this iterative procedure including extensive washing steps, sequencing lasts several hours to days. In case of Illumina and SOLiD sequencing the four differently labeled nucleotides are flushed over the glass slide in parallel, while a sequential flooding of non-labeled native nucleotides occurs during 454 and Ion PGM sequencing. In the former case incorporation of nucleotides is detected based on specific fluorescent labels attached to the nucleotide, in the latter case products of the enzymatic nucleotide incorporation reaction are detected, i.e., proton or pyrophosphate release. While proton release can be directly measured as pH change by the semiconductor chip of the Ion Torrent instruments (Merriman et al., 2012), the pyrophosphate signal is further converted into a light signal via subsequent reactions including the enzyme luciferase (Ronaghi et al., 1998). The generation of a light signal has led to the term “pyrosequencing” for this technology.

The different strategies of adding nucleotides to the DNA template strand affect sequence read length. During Illumina and SOLiD sequencing, a blocking group at each of the (oligo-) nucleotides prevents the addition of more than one molecule, so that the sequence is increased by one (oligo-) nucleotide at each step and the full read length is determined by the number of sequencing cycles performed (Bentley et al., 2008). In contrast, 454 and Ion PGM sequencing result in sequence reads of variable length. Due to the fact that the four different nucleotides are applied in a specified sequential order, a variable number of nucleotides is incorporated after four cycles, depending on the sequence of the respective library molecules. Several nucleotides are incorporated within the same cycle if the DNA template strand shows a homopolymeric region. This comes along with a proportional increase in signal strength, so that signal intensity is used to calculate the number of incorporated nucleotides (Margulies et al., 2005).

### Specifications of the different sequencing platforms

Major progress has been made during the last years with regard to sequence read length and output (number of reads per run) by technically improving the instruments, the chemistry and base-calling algorithms. A compilation of current specifications as given in Table 2 is useful to assess and compare the potential of the different instruments. The presented data were taken from the websites of the sequence providers. It should be kept in mind that those data were generated under optimum conditions. The specifications may not be met when more difficult sampling material is sequenced, e.g., libraries with more extreme GC content or of sub-optimal fragment length.

**Table 2:** **Data output of currently commercially available next generation sequencing platforms**.

**Company platform**	**No of units on sequencing support**	**Sequencing run conditions and read length^a^**	**Sequencing run time^b^**	**Maximum data output per run^c^**	**Maximum output in mio reads^d^**
**ROCHE**					
454 FLX+	1 PTP with gaskets to separate 2, 4, 8 or 16 regions	FLX (modal 450 bp, max. 600 bp)	10 h	450 Mb	1 per PTP (0.7 for amplicons)
		FLX+ (modal 700 bp, max. 1000 bp)	23 h	700 Mb	1 per PTP (0.7 for amplicons)
454 GS Junior Titanium	1 PTP	~450 bp	10 h	35 Mb	0.1 per PTP (0.07 for amplicons)
**ILLUMINA**					
HiSeq 2000/2500 (High output mode) V3 kits	8 lanes per flow cell, 1 or 2 flow cells per run	36 bp	2 days	95–105 Gb	165–185 per lane
		2 × 50 bp	5.5 days	270–300 Gb	
		100 bp	5 days	270–300 Gb	
		2 × 100 bp	11 days	540–600 Gb	
HiSeq 2000/2500 (High output mode) V4 kits	8 lanes per flow cell, 1 or 2 flow cells per run	36 bp	29 h	128–144 Gb	250 per lane
		2 × 50 bp	2.5 days	360–400 Gb	
		2 × 100 bp	5 days	720–800 Gb	
		2 × 100 bp	6 days	900–1000 Gb	
HiSeq 2500 (Rapid run mode) V3 kits	2 lanes per flow cell (not independent), 1 or 2 flow cells per run^e^	36 bp	7 h	18–22 Gb	125–150 per lane
		2 × 50 bp	16 h	50–60 Gb	
		2 × 100 bp	27 h	100–120 Gb	
		2 × 150 bp	40 h	150–180 Gb	
HiSeq X ten^f^	1 or 2 flow cells	2 × 150 bp	<3 days	1.6–1.8 Tb	3000 per flow cell
miSeq, V2 kits	1 lane, 1 flow cell	36 bp	4 h	540–610 Mb	12–15 per flow cell
		2 × 25 bp	5.5 h	750–850 Mb	
		2 × 150 bp	24 h	4.5–5.1 Gb	
		2 × 250 bp	39 h	7.5–8.5 Gb	
miSeq, V3 kits	1 lane, 1 flow cell	2 × 75 bp	24 h	3.3–3.8 Gb	22–25 per flow cell
		2 × 300 bp	55 h	13.2–15 Gb	
NextSeq 500 (High output mode)	4 lanes (not independent), 1 flow cell^e^	75 bp	11 h	25–30 Gb	400 per flow cell
		2 × 75 bp	18 h	50–60 Gb	
		2 × 150 bp	29 h	100–120 Gb	
NextSeq 500 (Mid output mode)	4 lanes (not independent), 1 flow cell^e^	2 × 75 bp	15 h	16–20 Gb	130 per flow cell
		2 × 150 bp	26 h	32–39 Gb	
**LIFE TECHNOLOGIES**					
SOLiD 5500xl	2 × 6 lanes	75 bp	5 days	160 Gb	160 per lane
		75 bp + 35 bp	8 days	220 Gb	
		60 bp + 60 bp	8 days	260 Gb	
SOLiD 5500xl W	2 × 6 lanes	50 bp	4 days	160 Gb	265 per lane
		75 bp	5 days	240 Gb	
		2 × 50 bp	8 days	320 Gb	
Ion PGM, 314 chip v2	1 Chip	200 bp mode	2.3 h	30–50 Mb	0.4–0.55 per chip
		400 bp mode	3.7 h	60–100 Mb	
Ion PGM, 316 chip v2	1 Chip	200 bp mode	3.0 h	300–600 Mb	2–3 per chip
		400 bp mode	4.9 h	600 Mb–1 Gb	
Ion PGM, 318 chip v2	1 Chip	200 bp mode	4.4 h	600 Mb–1 Gb	4–5.5 per chip
		400 bp mode	7.3 h	1.2–2.0 Gb	
Ion Proton, PI chip	1 Chip	200 bp mode	2–4 h	Up to 10 Gb	60–80 per chip
**PACIFIC BIOSCIENCES**					
PacBio RS II	Up to 16 SMRT cells	C2/P4 chemistry, mean read length ~8000 bp	2–3 h per cell	400 Mb per cell	0.05 per SMRT cell

a “2 ×” refers to paired end runs; more run conditions in the given range are possible for Illumina instruments.

b Sequencing time does not include library amplification, except for the MiSeq and NextSeq platforms.

c Output for 2 flow cells per run in case of the Illumina HiSeq systems.

d The two reads of a paired end read are counted as one paired end read here.

e Lanes can only be independently loaded with different libraries if cluster amplification is done on the cBot.

f Not yet available, dedicated to human genome sequencing.

The SOLiD and Illumina HiSeq sequencers generate the largest amount of data per run at the lowest costs per base. Soon Illumina HiSeq instuments will produce up to 1000 Gb per run. At the same time, these platforms generate the shortest reads. In particular the very short SOLiD sequence reads are mostly used for resequencing and transcriptomics projects, in which reads can be mapped to known genomes, but not frequently in *de novo* sequencing projects. Between 8 and 11 days are needed to perform a run with maximum data output on these instruments. Illumina has developed strategies during the last years to reduce run time, resulting in the upgrade of the HiSeq 2000 instrument to HiSeq 2500. The upgrade allows sequencing in rapid run mode, which produces a smaller amount of data (approximately 25–30% of data compared to a so-called “high-output” run) within hours to 2 days, depending on the desired read length. The upgrade came along with an increase in maximum read length from 100 to 150 bp in rapid run mode.

The Illumina MiSeq platform was launched in 2011. This platform produces 22–25 million reads with a maximum length of 300 bp when using the new V3 chemistry. The costs per sequenced base are higher compared to the HiSeq instrument. However, the longer read length in combination with the lower read number can be of particular interest for amplicon sequencing projects. It is also very suitable for small scale metagenomics projects or initial sample evaluation prior to deep sequencing on a HiSeq. The newest releases from Illumina are the NextSeq 500 platform, which performs at intermediate scale in terms of output, read length, and costs per base compared to HiSeq and Miseq, and the HiSeqX ten, a package of 10 HiSeq sequencers, which allow even higher throughput than the HiSeq2500 in shorter time.

The 454 sequencer was the first commercially available NGS instrument (since 2005). In comparison to Illumina and SOLiD platforms, it generates longer reads (modal read length 750 bp, average read length 700 bp) in a shorter run time (1 day) using FLX+ chemistry. The total output per run of this platform is clearly lower in terms of reads (1 million) and bases (700 Mb). The higher costs per base are a major reason why its use is meanwhile often replaced by the aforementioned platforms, in particular in projects in which coverage is more important than read length, as it is for instance the case in transcriptomics projects, some metagenomic projects or amplicon sequencing projects. Also Roche has released a smaller-scale benchtop sequencing instrument, the 454 GS Junior (available since 2009). This sequencer produces approximately 100,000 reads per run with a modal read length of 450 bp, comparable to the read length obtained with the FLX+ platform when run with FLX chemistry instead of FLX+ chemistry.

The Ion Torrent PGM sequencer is available on the market since the end of 2010. Sequencing on this platform is done using semiconductor chips of different scale, which allow to sequence between 0.4 and 5.5 million reads. Read length on this platform increased successively from approximately 100 bp to meanwhile 400 bp. Sequencing on Ion instruments is very fast, taking only a couple of hours. The Ion Proton is a larger-scale instrument that produces 10-fold more bases per run using the Ion PI chip. A larger scale chip (Ion PII) is announced for this platform. In terms of sequencing costs per base, the Ion PGM ranges in between 454 and Illumina/SOLiD technologies.

### Paired end sequencing and mate pair libraries

Most sequencers allow sequencing of library fragments from both ends. A corresponding reverse read can be assigned to each individual forward read in Illumina and SOLiD paired end sequencing mode. Since the average size of the library molecules is known, the distance between forward and reverse read is also known. This information is very helpful when performing assembly or read mapping. Paired end reads can also be used to improve sequence quality of short amplicons when overlapping reads are generated. Paired end sequencing is also possible on the Ion Torrent instruments and protocols are available, but this sequencing mode is not yet officially supported by the company.

**Figure 2:**
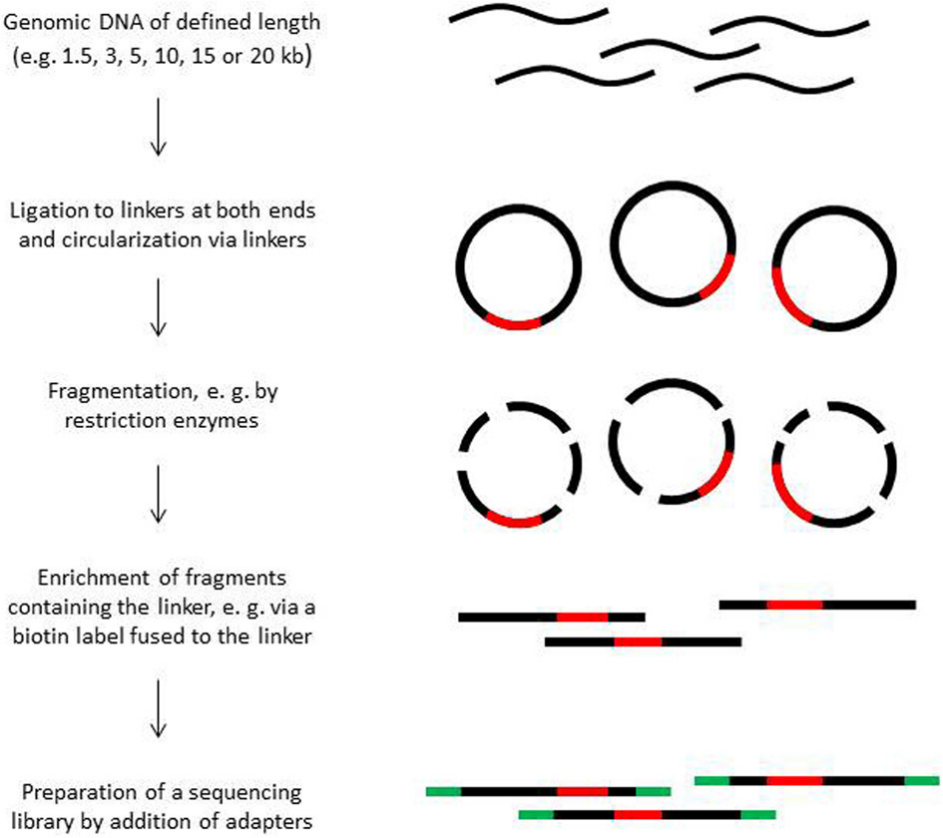
**Construction of mate pair libraries**.

Paired end sequencing can be done for library fragments of up to approximately 800 bp. However, in *de novo* sequencing projects read pairs spanning even larger distances are helpful to bridge longer repetitive regions (Mavromatis et al., 2012). Paired sequence reads spanning distances between 1.5 and 20 kb can be obtained from mate pair libraries. The construction principle of such libraries is shown in Figure 2. Mate pair libraries are sequenced in paired end run mode if available. On 454 instruments, mate pair libraries can also be sequenced; the reads will contain sequence information from both ends, separated by the linker sequence somewhere in the middle of the read.

The construction of mate-pair libraries is quite expensive not only monetarily, but also with regard to the amount of input material. Mate pair libraries spanning long distances need 15–20 μ g of high molecular weight DNA of which most is lost during the enrichment step of the end-to-end ligated fragments. A certain percentage of library molecules will consist of molecules in which one of the two ends is only represented by a few nucleotides due to the random fragmentation process of the circularized molecules. Such short fragments cannot be assembled with certainty and are discarded. Moreover, the library construction procedure is not free of bias, which can negatively affect assembly, and the diversity of fragments can be rather low, in particular when the amount of input material is limited. When sequencing organisms with small genomes such as bacterial strains, a few hundred thousand reads are usually sufficient to cover the diversity of constructs present in a library. The use of sequencing platforms that produce long reads such as the PacBio instrument appears to become an interesting alternative to mate pair library sequencing.

## Single molecule sequencing

Despite the fact that single molecule sequencing approaches are mostly still under development, they have already been described in diverse review articles (e.g., Gupta, 2008; Xu et al., 2009; Schadt et al., 2010; Treffer and Deckert, 2010; Niedringhaus et al., 2011; Pareek et al., 2011; Zhang et al., 2011; Liu et al., 2012; Morey et al., 2013). Currently, the instrument from Pacific Biosciences is the only commercially available platform. Helicos Biosciences, the company that actually released the first single molecule sequencer, vanished from the market in 2012. The major goals that guide the development of single molecule sequencing platforms are longer read length, higher throughput, higher accuracy, faster turnaround time and lower costs per base (Schadt et al., 2010). It remains to be seen how well all these specifications can be met by one single instrument and which of the different systems currently under development will successfully establish on this highly competitive market.

### Single molecule sequencing with the PacBio RS

The sequencing technology of the PacBio RS is described in detail in the above mentioned reviews about single molecule sequencing and in articles that introduce this sequencing system to the scientific community (Eid et al., 2009; Korlach et al., 2010). In brief, the principle of this single molecule real-time (SMRT) technology is to attach a DNA polymerase molecule on the bottom surface of a zero-mode waveguide detector (ZMW). The ZMW enables the detection of fluorescence of individual nucleotides that are incorporated by the polymerase into a single complementary DNA strand during the synthesis process. Each type of dNTP has a unique fluorescent label that is cleaved off during DNA synthesis. The ZMWs can be considered as densely arranged nano-chambers in a perforated metal film on top of a glass surface, enabling the parallelization of the sequencing process in 150,000 ZMWs within a SMRT cell (Levene et al., 2003). The ZMWs are scanned for fluorescent signals by a confocal imaging system, resulting in movies of up to 120 min or even 240 min in the near future that document the successive incorporation of nucleotides, from which the sequence is deduced. Nucleotide incorporation occurs continuously without intermittent washing steps, which accelerates sequencing substantially compared to second generation sequencing systems.

Initially, the DNA synthesis reaction could be monitored only in half of the ZMWs on the PacBio RS system at the same time, but a recent upgrade to RS II enables parallel recording of all ZMWs. However, not all ZMWs produce usable reads, so that the expected number of reads for a SMRT cell is approximately 50,000 for the RS II system. Currently, sequencing is done with the C2/P4 chemistry, but will soon be changed to C3/P5, which will support longer movies and thus the generation of longer reads. The mean read length of the instrument is around 8000 bases, probably increasing to 8500 bases with the new chemistry. A maximum read length of more than 20 kb was observed in different projects, reads of 16 kb are regularly obtained in runs with good quality libraries. In comparison to other sequencing platforms, read length and sequencing time are superior, while output per run is clearly lower and the costs per base are rather high. However, the costs for one SMRT cell are relatively low. These specifications suit in particular bacterial genome sequencing projects.

To improve sequence read quality, a circular consensus sequencing (CCS) strategy was developed. It is based on the fact that PacBio libraries have a circular molecule structure, referred to as SMRTbell template (Travers et al., 2010). These libraries are constructed by ligating hairpin loop adapters to the DNA fragments. The circular structure allows a continuous and repeated sequencing of sense and antisense strand, which can be used to generate single consensus reads with very high accuracy (>99%). The accuracy comes at the expense of read length, since the maximum recording time is limited. Thus, the length of the library molecules determine how often a strand is sequenced within the given time. The higher the desired accuracy of the reads the shorter the reads should be. It depends on the project whether high accuracy reads or longer reads are more valuable. In *de novo* genome sequencing projects the length of the reads is of higher relevance to support genome assembly. In contrast, high-accuracy single consensus sequencing can be useful in metagenomic and especially in amplicon sequencing projects, as higher accuracy prevents an overestimation of biological diversity due to sequencing errors.

### Future single molecule sequencing technologies

Nucleotide identification of currently available sequencing platforms is mostly based on optical systems that detect incorporation of fluorescently labeled nucleotides or reaction products during DNA synthesis. Future sequencing methods aim at real-time label-free sequencing, e.g., by direct analysis of the DNA molecule using electron microscopic techniques, scanning tunneling microscopy and spectroscopy, or analysis by Raman spectroscopy. Nanopore sequencing is another strategy that has gained much attention and has already been addressed in a couple of reviews (Bayley, 2006; Branton et al., 2008; Xu et al., 2009; Timp et al., 2010; Maitra et al., 2012). The different nanopore sequencing strategies that are under development enable individual base detection based on the measurement of conductivity changes across a lipid membrane while a DNA fragment is pulled through a nano-scale pore by an electric current. Conductivity changes are nucleotide-specific, enabling the identification of nucleotides as they traverse the pore. Biological nanopores are either constructed from engineered proteins, e.g., α-hemolysin (originally from *Staphylooccus aureus*) or MspA (*Mycobacterium smegmatis* porin A), or are entirely synthetic, e.g., graphene (Schadt et al., 2010; Thompson and Milos, 2011; Maitra et al., 2012). One of the major challenges in nanopore sequencing is reliable signal detection of each individual nucleotide at the high speed at which the DNA molecule traverses the pore and against a background of stochastic alterations in translocation rate (Branton et al., 2008; Morey et al., 2013).

As single molecule sequencing technologies do no longer depend on a PCR amplification step for signal detection, they overcome any bias introduced during emPCR or bridge PCR as well as dephasing problems (see Section Error Accumulation toward the End of Reads) that result in signal decay, which largely limits read length of current second generation instruments. These advantages come along with a higher sequencing error rate in individual reads, as errors cannot be compensated by the consensus read-out of clonal molecules in a cluster or on a bead. Future improvements of the sequencing technologies and the generation of consensus sequences, as explained for the PacBio instrument, have the potential to compensate these errors.

## Sequencing errors

### Estimated error rates of second generation sequencing platforms

In comparison to Sanger sequencing, NGS technologies are known for higher error rates and different types of errors in the generated sequence reads. A direct comparison of error rates from different sequencing platforms and studies is difficult due to differences with regard to the sequenced sample material, the library preparation method, data filtering, and error calculation methods, and the fact that reads of different length (not necessarily the maximum possible length of a platform) are analyzed. Nevertheless, some values are compiled and provided as Table S1 for orientation. They are mostly in the range of 0.4–1% for Roche 454, Illumina and the Ion PGM platforms. Clear differences between these platform are not evident from the data. The quality of Ion PGM data, which is discussed quite controversially in the literature, is often slightly lower in direct comparison to Illumina and 454 platforms (Liu et al., 2012; Loman et al., 2012; Quail et al., 2012; Jünemann et al., 2013; Perkins et al., 2013). Read quality of HiSeq data was mostly reported to be slightly better compared to GAIIx data (Meacham et al., 2011; Minoche et al., 2011; Quail et al., 2012). The error profiles for the Illumins GA, HiSeq, and MiSeq instruments remain principally the same (Minoche et al., 2011; Quail et al., 2012). The quality of sequencing data from different 454 platforms appears to be similar. Likewise differences in dependence of the used chemistry or the analyzed library type (shotgun or amplicon) are not evident.

Substantial effort has been made to identify different types and sources of sequencing errors with the aim to reduce these either during the sequencing process or afterwards by applying improved analyses and correction algorithms. Some sequencing errors are observed on all sequencing platforms, while others are platform-specific. The following discussion about sequencing errors is largely focused on two sequencing platforms, 454 and Illumina, since error evaluation has been most intensively done for these platforms and these are the most frequently used platforms.

### Error distribution within reads of a library

If the distribution of errors among 454 reads would be completely random, an error rate of 0.5% would mean that each read of 500 bp has on average 2.5 errors. But sequencing errors occur only in a certain percentage of reads; most studies report around 70% error-free reads (Huse et al., 2007; Kunin et al., 2010; Niu et al., 2010; Prabakaran et al., 2011; Niklas et al., 2013). Huse et al. (2007) observed that many of the erroneous reads in an amplicon dataset were characterized by the simultaneous presence of ambiguous base calls and explained this with multitemplated beads that carry similar library fragments.

In Illumina datasets, an increasing number of errors is observed in a successively decreasing number of reads (Dohm et al., 2008; Hillier et al., 2008; Nguyen et al., 2011). The percentage or error free reads was reported to be 57% for the GAIIx platform and 76% for the MiSeq platform in two available reports (Hillier et al., 2008; Quail et al., 2012). During paired end sequencing, the forward read was usually of slightly better quality than the reverse read (Quail et al., 2008; Minoche et al., 2011).

### Types of sequencing errors and their frequency

Insertions are the most frequent type of error during 454 sequencing (e.g., Margulies et al., 2005; Prabakaran et al., 2011; Vandenbroucke et al., 2011; Skums et al., 2012; Niklas et al., 2013). Several studies have reported deletions to be the second-most frequent type of error, followed by substitution errors (Huse et al., 2007; Gilles et al., 2011; Schloss et al., 2011; Niklas et al., 2013). The majority of indel errors occurs in homopolymeric regions (Margulies et al., 2005; Huse et al., 2007; Rozera et al., 2009; Kunin et al., 2010; Gilles et al., 2011; Shao et al., 2013). The longer the homopolymeric region, the higher the probability of an indel error and the lower the quality scores of the bases toward the end of this region (Quinlan et al., 2008; Luo et al., 2012b; Skums et al., 2012; Niklas et al., 2013). Indel errors are explained by the underlying sequencing principle. The preciseness of the proportionality of the detected light signal decreases with increasing number of identical bases (Margulies et al., 2005). Due to an analogous sequencing principle, the Ion PGM sequencer shows a similar error profile, dominated by indel errors in homopolymeric regions and clearly less substitution errors (Loman et al., 2012; Merriman et al., 2012; Bragg et al., 2013).

In contrast, substitution errors are the most frequent error type in Illumina sequencing (Dohm et al., 2008; Hillier et al., 2008; Hoffmann et al., 2009; Minoche et al., 2011; Nguyen et al., 2011) and for SOLiD sequencers (Shendure and Ji, 2008; Ratan et al., 2013). For the Illumina platform, Nguyen et al. (2011) identified 79–88% of all errors as substitution errors. Hillier et al. (2008) reported a 3.7-fold higher substitution error rate than indel error rate. Deletions are more frequent than insertions and insertions are likely to occur in homopolymeric regions (Dohm et al., 2008; Minoche et al., 2011). The lower rate of indel errors compared to 454 sequencing is achieved by the terminal blocking strategy during the sequencing process, which allows the incorporation of only one base per sequencing cycle, so that a homopolymeric region is sequenced base by base.

### Error accumulation toward the end of reads

Sequencing errors accumulate toward the end of reads, along with decreasing quality of the called bases. This is well known for Illumina reads, but has also been reported for 454 and Ion PGM data (Campbell et al., 2008; Lind et al., 2010; Schröder et al., 2010; Huse and Welch, 2011; Schloss et al., 2011; Loman et al., 2012; Bragg et al., 2013; Perkins et al., 2013). This accumulation of errors is the result of a decreasing signal-to-noise ratio during the sequencing process, which largely determines the maximum read length of all sequencing platforms.

Errors in 454 reads occur more likely beyond base 200–300 under FLX run conditions on the FLX and the GS Junior platform (Campbell et al., 2008; Gilles et al., 2011; Schloss et al., 2011; Niklas et al., 2013). In particular substitutions and ambiguous base calls accumulate (Gilles et al., 2011). Such an error profile is the result of a loss of synchronism during the sequencing process on the multitemplated beads. Even though the basecalling software accounts for this artifact and reads are trimmed, it does not fully eliminate these effects (Margulies et al., 2005; Gilles et al., 2011). Another reason for a decreasing signal-to-noise ratio toward the end of a read is signal drooping due to premature termination of the sequencing process on templates. This was reported for Ion PGM sequencing (Merriman et al., 2012; Golan and Medvedev, 2013).

In Illumina reads, an accumulation of errors toward the end mainly affects long reads. It becomes obvious in the last third to fourth of 100 or 150 bp reads (Dohm et al., 2008; Claesson et al., 2010; Minoche et al., 2011; Nakamura et al., 2011; Liu et al., 2012). The result of this accumulation are lower overall quality values for longer reads. Also on Illumina platforms, the decreasing signal-to-noise ratio is largely a problem of signal dephasing during the sequencing process (Erlich et al., 2008; Kircher et al., 2009; Metzker, 2010; Schadt et al., 2010). Dephasing occurs when part of the clonal fragments in a cluster on the flow cell lag behind or are advanced compared to the overall sequencing procedure. The signal-to-noise ratio also decreases when the fluorescent label is not efficiently cleaved from the nucleotides added in the previous cycle (Dohm et al., 2008), and due to fluorescent dye decay during the sequencing process over several days (Kircher et al., 2009).

### Sequencing error context dependence

Substitution errors in Illumina reads were analyzed in more detail to identify possible error sources (Dohm et al., 2008; Meacham et al., 2011; Minoche et al., 2011; Nakamura et al., 2011; Nguyen et al., 2011; Abnizova et al., 2012; Luo et al., 2012b; Quail et al., 2012). Certain types of substitutions were found to occur more frequently than others and accumulate at specific positions. They are sequence context dependent, for instance after G-rich regions (Dohm et al., 2008; Minoche et al., 2011). Moreover, many substitution errors occur strand-specific, i.e., either predominantly in reads that cover a genomic region in forward direction or in those of reverse direction (Meacham et al., 2011; Nguyen et al., 2011). Such errors can be identified during data assembly or read mapping based on their strand-specificity and the fact that they are associated with low quality values for the respective erroneous base (Minoche et al., 2011). Abnizova et al. (2012) observed that the correct base was frequently detected with the second most intensive sequencing signal at erroneous positions, providing a possibility for correction. That errors tend to accumulate at specific positions within a genome was also observed for SOLiD data (Meacham et al., 2011).

### Evenness of read coverage and GC bias

Early NGS studies already reported uneven read coverage when Illumina reads were mapped to existing genomes (Dohm et al., 2008; Hillier et al., 2008). The extent of this variation appears to vary largely from only 2- or 4-fold (Dohm et al., 2008; Minoche et al., 2011) to more than 100-fold (Harismendy et al., 2009). It can also occur in SOLiD, 454 and Ion PGM datasets (Suzuki et al., 2011; Meglecz et al., 2012; Merriman et al., 2012; Balzer et al., 2013; Gori et al., 2013; Ratan et al., 2013). In comparative studies, each platform produced a specific coverage pattern (Harismendy et al., 2009; Quail et al., 2012; Rieber et al., 2013). Depending on the coverage with which a sample is sequenced, this bias can result in gaps and affect quantitative assessments, e.g., in metagenomic or (meta)transcriptomic studies (Tariq et al., 2011; Gori et al., 2013).

A detailed analysis revealed an underrepresentation of reads in AT-rich regions (Bentley et al., 2008; Dohm et al., 2008; Hillier et al., 2008; Harismendy et al., 2009; Kozarewa et al., 2009; Minoche et al., 2011; Quail et al., 2012) and GC-rich regions (Bentley et al., 2008; Kozarewa et al., 2009; Quail et al., 2012; Ratan et al., 2013). It is the GC content of the complete library molecule and not only of the sequenced region that affects GC bias (Benjamini and Speed, 2012).

PCR steps were identified as a major cause introducing GC bias (Hillier et al., 2008; Aird et al., 2011; Quail et al., 2012). Standard Illumina and Ion PGM library preparation protocols include a PCR amplification step prior to bridge PCR or emPCR. To reduce GC bias, PCR free protocols have been developed for Illumina library construction (Kozarewa et al., 2009; Mamanova and Turner, 2011) and have meanwhile also been implemented in dedicated Illumina kits. Since PCR-free library preparation methods are problematic when the available input material is limited, PCR protocols were also optimized, as well as other library preparation steps that may introduce such bias (Van Dijk et al., 2014). High cluster densities on the Illumina flow-cell were also discussed to suppress GC-rich reads (Aird et al., 2011). Error correction algorithms were developed and can be applied to account for GC-bias in projects where quantitative information is inferred from the sequencing data such as transcriptomic studies (Hansen et al., 2010; Li et al., 2010; Benjamini and Speed, 2012).

### Duplicate reads

Another artifact that has been reported in particular for 454 sequencing data is the occurrence of duplicate reads in shotgun (meta-)genomic sequencing projects. These start at the same base position and, depending on the strictness of the definition, are fully identical or different in only few positions and/or read length. Such sequence reads can be true duplicates that arise when genomic DNA is sequenced at very high coverage, or they are artificial duplicates. The source of this type of error is not fully known. It was speculated that duplicates are generated during emPCR, when amplified DNA is attaching to empty beads (Briggs et al., 2007). However, emPCR is also used to amplify library fragments during Ion PGM sequencing, but duplicate reads appeared not to be a major problem in one study in which this issue was specifically assessed (Bragg et al., 2013).

The analysis of several metagenomic sequencing projects revealed between 10 and 45% of duplicate reads (Gomez-Alvarez et al., 2009; Niu et al., 2010; Balzer et al., 2013). Duplicate reads can affect quantitative data analyses, e.g., species or gene abundance analyses in metagenomic studies. To identify and remove duplicates, software tools such as cd-hit-454 (Niu et al., 2010), 454 Replicate Filter (Gomez-Alvarez et al., 2009), PyroCleaner (Mariette et al., 2011), the duplicate removal tool of the GATK package (McKenna et al., 2010), or JATAC (Balzer et al., 2013) can be applied. Criteria that define artificial duplicates can be defined in such software tools. Nevertheless, some true duplicate reads may also be eliminated by these filters. The percentage of true duplicates among all identified duplicates can vary largely between 2 and 72% (Niu et al., 2010).

### Reproducibility across runs and between regions or lanes

The overall reproducibility between 454 runs and samples from different regions of the picotiter plate is usually high (Vandenbroucke et al., 2011; Niklas et al., 2013). However, variation in error rates, in particular for indel errors, was seen between different 454 sequencing runs (Gilles et al., 2011; Prabakaran et al., 2011; Shao et al., 2013). Variation in terms of read composition of a sample may also occur, as observed in a study in which the same 16S rRNA gene PCR products were sequenced at different sequencing centers and in different runs (Schloss et al., 2011). A similarity analysis of the datasets revealed a clustering according to sequencing centers and, to lesser extent, to runs.

For Illumina, some studies report variation between runs and from lane to lane, e.g. with regard to sequencing errors (He et al., 2010; Aird et al., 2011; Nguyen et al., 2011; Chen et al., 2013), but also in this case it seems not to be a consistent problem (Abnizova et al., 2012; Benjamini and Speed, 2012). Nguyen et al. (2011) reported that variation with regard to sequencing errors largely diminished after data quality filtering. Highly reproducible results were also obtained in a study by Caporaso et al. (2012) across lanes and even on different platforms (i.e., HiSeq 2000 and MiSeq), showing that cross-platform data handling is possible (Bokulich et al., 2013).

It will depend on the project whether possible variation in sequencing performance is acceptable or will negatively affect results and conclusions. It can be a relevant issue when highly similar samples are comparatively analyzed, e.g., in amplicon sequencing projects. To identify method related variation in such critical studies, the inclusion of a standardized reference sample is highly recommended (Schloss et al., 2011; Bokulich et al., 2013).

### Sequencing errors of the PacBio RS system

Sequencing errors of PacBio single reads are reported in the range of 13–20% (Thompson and Milos, 2011; Quail et al., 2012) but this high error rate can be reduced to 1% or less by CCS (Metzker, 2010). Sequencing errors on the PacBio system are mostly insertions and deletions (Eid et al., 2009). During single molecule sequencing, dephasing is not an issue, so that errors do not accumulate toward the end of the reads. Moreover, sequencing errors appear not to be sequence context specific (Carneiro et al., 2012; Koren et al., 2012) contributing to the high consensus accuracy that can be achieved when sequencing is done with high coverage (>20-fold) or by using the CCS strategy. Good performance was reported in difficult to sequence regions and GC-rich samples, resulting in more even coverage (Quail et al., 2012; Ross et al., 2013; Shin et al., 2013).

### Compensating and correcting sequencing errors

Once the types and sources of sequencing errors are known, different strategies and tools can be developed to compensate and correct errors. As a general strategy, accuracy is improved by sequencing with high coverage, usually 20- to 60-fold, depending on the sequencing purpose (Margulies et al., 2005; Voelkerding et al., 2009; Luo et al., 2012b). Also, the combination of sequencing data generated from different sequencing platforms with different error profiles was suggested and has been applied to identify and eliminate sequencing errors (Nakamura et al., 2011; Koren et al., 2012). These strategies are effective in *de novo* genomic sequencing and resequencing projects, but they are of limited use in metagenomic or metatranscriptomic studies that deal with biological variation. Each different read can represent a distinct genotype in such studies or is the result of a sequencing error. Sophisticated methods are needed to distinguish between natural sequence variation and sequencing errors in order not to overestimate diversity.

One way to reduce error rates is to apply alternative basecallers that show superior performance compared to the standard basecalling algorithms (e.g., Ledergerber and Dessimoz, 2011; Das and Vikalo, 2013; Golan and Medvedev, 2013). However, their application is often limited, as it comes along with a transfer of massive amounts of raw signal data from the sequencing service center to the customer and the need for high computational power to perform basecalling, in particular for large Illumina datasets.

In order to improve data quality after basecalling, filtering algorithms were developed. Such filters discard reads with low-quality bases or with uncalled/ambiguous bases, or they clip the lower quality 3'-ends of reads. Many of these filters use the information contained in quality values that are calculated for each base during the base calling process. Minoche et al. (2011) studied the effect of different filtering methods on Illumina data and could reduce the error rate to <0.2% by eliminating approximately 15–20% of the low-quality bases, mostly via 3'-end trimming. Nguyen et al. (2011) reported a 5-fold decrease of the error rate by applying a filter that eliminated reads with low quality bases (<Q30; i.e., with 0.1% likelihood of a false basecall), which resulted in a loss of 24–35% of sequence reads. It has to be kept in mind that low quality bases are to certain extent localized in specific regions of a genome. Discarding such reads can result in a more uneven coverage, introducing potential bias in quantitative studies (Minoche et al., 2011; Nakamura et al., 2011).

An alternative strategy to read clipping and exclusion of low quality reads is error correction. Several tools (e.g., Coral, HiTEC, Musket, Quake, RACER, Reptile, or SHREC) have been developed for this purpose, in particular for the correction of substitution errors in Illumina data (Ilie and Molnar, 2013; Liu et al., 2013; Yang et al., 2013). Some of these tools (Coral, HSHREC, KEC, and ET) have implemented indel correction algorithms and are thus suited for the analysis of 454 and Ion PGM data (Salmela, 2010; Salmela and Schröder, 2011; Skums et al., 2012). Error correction methods make use of the high sequence coverage in order to identify and correct errors. Moreover, most algorithms take into account the quality scores given for the individual bases and/or analyze the neighboring contextual sequence information. The application of error correction tools has been proven useful in *de novo* genome sequencing projects, resequencing and amplicon sequencing projects (e.g., Skums et al., 2012; Yang et al., 2013). At the same time, Yang et al. (2013) pointed out a need for improved algorithms, in particular for non-uniform data sets, such as metagenomic or (meta-)transcriptomic data. A strategy that can be applied in metagenomics studies to correct sequencing errors is the generation of overlapping paired end reads that are assembled prior to further analyses (Zhou et al., 2011; Masella et al., 2012; Eren et al., 2013).

## Metagenomic sequencing of the plant associated microbiota

### Sequencing and analysis strategies for metagenomics studies

The optimal sequencing strategy for a metagenomics project will largely depend on the aim of the project. For a functional description of a microbial community, the Illumina HiSeq sequencing platform will be a good choice due to the low costs per sequenced base, which allows sequencing to high depth in order to gain as much information as possible, even from less-abundant microorganisms that may nevertheless play important roles for ecosystem functioning. Initially, the rather short read length of this platform was considered to be a critical issue (Wommack et al., 2008), but it appears that this is not necessarily a problem. A comparative study of a metagenomic analysis based on 454 and Illumina reads revealed that assembled data derived from both methods reflected the genomic composition of the sample equally well, with the Illumina dataset showing even a slightly better assembly result (using a 5-fold higher volume of data) (Luo et al., 2012b). Annotation of unassembled reads was slightly better for the longer 454 reads. In general, short reads will not allow the generation of a high number of large contigs, in particular for complex samples. As an example, assembly success for a metagenomic sample from the soybean phyllosphere microbiota, which showed medium complexity, was only moderate. The assembly of approximately 1 mio 454 reads with a mean read length of 235 bp resulted in 140,000 contigs with a mean length of 276 bp and left 30% of the reads unassembled. The largest contig had a length of 12,888 bp (Delmotte et al., 2009). In another study with datasets from complex freshwater microbial communities between 50 and 60% of 454 and Illumina reads remained unassembled (Luo et al., 2012b). Despite this moderate success, gene prediction or identification of protein domains is possible. This is even the case for unassembled short reads, though it becomes more difficult when no close homolog is present in the reference database (Scholz et al., 2012; Luo and Moran, 2013). Moreover, annotation of several million unassembled short reads can become a very time-consuming step, depending on the algorithm that is used.

An alternative to assembly and/or direct annotation of short sequence reads is the mapping of reads to existing genomes. The prerequisite for this strategy is that the genomes of the organisms of interest have been genome sequenced. This is currently still a limiting factor (Weinstock, 2011), although the entries in public databases are much more strongly growing since NGS technologies became available. Currently, there are nearly 3000 complete genome sequences of microorganisms deposited in the NCBI database and genomic information of approximately 16,000 microorganisms is available as scaffolds or contigs. It can be a very valuable step to enrich, isolate and sequence the dominant community members, as it is for instance done in the Human Microbiome Project (Turnbaugh et al., 2007), or was already done for 21 bacterial isolates from the *Populus* rhizosphere (Brown et al., 2012). Such attempts will be of value for diverse studies of plant associated microorganisms, as the plant associated microbiota appears to show certain degree of consistency in terms of colonizing taxa (Bulgarelli et al., 2012; Lundberg et al., 2012; Vorholt, 2012), so that stains sequenced in one study may support data analysis of another study using plants grown under different conditions or even different model plants. Thus, the generation of further individual genome sequences will improve data analysis of future metagenomics, metatransriptomics, and metaproteomics studies of plant-associated microorganisms.

As several microbial taxa remain unculturable, some metagenomic studies aim at the reconstruction of individual genomes to obtain information from these organisms. In such studies sequence read assembly is a key step and challenging due to the complexity and uneven composition of microbial communities (Scholz et al., 2012). Assembly will be most successful if the complexity of the microbial community is rather low and dominated by one or a few phylogenetically distinct bacterial taxa. Different studies have meanwhile demonstrated that genome reconstruction of individual members in metagenomic samples is possible, even when rather short Illumina reads are generated (Mackelprang et al., 2011; Albertsen et al., 2013).

Assembly success also depends on sequence read length and the coverage with which the genome(s) of interest are sequenced (Kunin et al., 2008; Schatz et al., 2010; Weinstock, 2011; Luo et al., 2012a); parameters that can be considered in the design of the sequencing strategy. In an *in silico* study, Luo et al. (2012a) demonstrated that a 20-fold coverage was sufficient to reconstruct the genome of a dominant member in a metagenomic sample and that a higher coverage did not substantially improve the assembly result. Strategies that are frequently applied in pure culture genome sequencing projects to improve assembly are the inclusion of longer reads, paired end reads or reads from mate pair libraries (Schatz et al., 2010). This strategy can also be useful in metagenomic sequencing projects. The combination of sequencing data from different platforms that generate reads of different lengths and with different error profiles was reported multiple times as a successful strategy to improve genome assembly of individual bacterial strains (Aury et al., 2008; Reinhardt et al., 2009; Koren et al., 2012). In particular the PacBio instrument holds potential to fulfill the need for long reads in order to bridge larger gaps or repetitive regions (English et al., 2012; Mavromatis et al., 2012). These strategies have not yet been widely applied in metagenomics projects, but it appears likely that they are of value (Niedringhaus et al., 2011).

Assemblies may also be improved by using new assembly strategies, e.g., a nested strategy, in which the short reads are assembled to longer reads in a first step, before those are further assembled. The *in silico* generation of Sanger-like reads from Illumina reads by filling the gaps between paired end reads can be done by searching for reads within the same library that fill the gap between a read pair or by constructing paired end libraries of successively decreasing insert length, which are searched for suitable paired end reads to close the gaps between those paired end reads that are contained in the library with the largest library molecules (Rodrigue et al., 2010; Nadalin et al., 2012; Ruan et al., 2013). This strategy may be of particular help to fill small gaps, i.e., of a distance smaller than the size of the largest library molecules, but will not help to bridge repetitive regions that are larger than the largest library molecules.

### Bioinformatics tools for metagenomic data analysis

The massive amount of sequence data that are generated in metagenomic projects demand new and efficient computational methods for data processing, analysis, and storage (Pop and Salzberg, 2008; Tautz et al., 2010). Substantial progress has been made in this field, as evident from the many different tools that are meanwhile available, e.g., for sequence read assembly, read mapping, or gene prediction (for an overview of available tools see for instance Voelkerding et al., 2009; Guazzaroni and Ferrer, 2011; Zhang et al., 2011; Thomas et al., 2012). New tools become available that are specifically designed for the analysis of metagenomic data, including assemblers such as MetaVelvet or Meta-IDBA (Peng et al., 2011; Namiki et al., 2012), annotation tools such as MG-RAST or CAMERA (Glass et al., 2010; Sun et al., 2011), tools for read mapping and alignment and for further data analysis, e.g., taxon identification and analysis of the microbial community composition based on phylogenetic marker genes (e.g., Stark et al., 2010; Scholz et al., 2012; Sunagawa et al., 2013). It would go beyond the scope of this review to discuss the diverse options for the analysis of metagenomic data along with the available software tools. Several recent reviews have addressed this aspect in detail (Kunin et al., 2008; De Filippo et al., 2012; Hunter et al., 2012; Logares et al., 2012; Scholz et al., 2012; Teeling and Glöckner, 2012; Davenport and Tümmler, 2013; Kim et al., 2013; Luo et al., 2013; Preheim et al., 2013; Segata et al., 2013).

Not only powerful software tools are required for the analysis of NGS data, but also high-performance computing capacity, in particular for large metagenomics datasets. This may pose a problem to research laboratories that are not specialized on NGS data analysis. Cloud computing, i.e., the rental of processing time on a computer cluster on demand over a network, is discussed and developing as a possible solution to this problem (Angiuoli et al., 2011; Wilke et al., 2011; Zhang et al., 2011; Dai et al., 2012; Nagasaki et al., 2013), though it has to be considered that this is often not free of costs and may pose security issues related to data transfer (Angiuoli et al., 2011; Hunter et al., 2012).

## Targeted gene sequencing of amplicons from metagenomic DNA

### Selecting the appropriate sequencing strategy for amplicon sequencing

Targeted sequencing approaches of metagenomic DNA are mostly applied to identify the members of microbial communities or to compare their composition in different samples. Diversity studies are usually based on the 16S rRNA gene as bacterial marker and 18S rRNA or ITS as fungal markers (Table S2), while functional marker genes are analyzed when microorganisms with specific metabolic functions such as chitin degradation are addressed (Cretoiu et al., 2012). Until now the fast majority of amplicon sequencing studies have been performed using 454 technology (Table S2), mostly due to the fact that this was the first available NGS platform and due to the relatively long reads, that can be obtained from this platform. However, a shift toward the Illumina platform is currently noticable. First studies were already performed on the GAIIx platform with 76 bp paired end reads and later on with longer paired end reads up to 150 bp, followed by analysis on the HiSeq instrument and recently also on the MiSeq platform (Claesson et al., 2010; e.g., Gloor et al., 2010; Hummelen et al., 2010; Caporaso et al., 2011, 2012; Jogler et al., 2011; Degnan and Ochman, 2012; Kozich et al., 2013; Bokulich et al., 2014). The generation of overlapping paired end reads is recommended on these platforms as it will help to minimize the error rate (Eren et al., 2013; Kozich et al., 2013). As outlined above, errors accumulate toward the end of the reads, so that they can be corrected if consensus reads are generated from the read pairs. In particular the MiSeq instrument is a suitable platform for such studies, as it produces reads with a length comparable to those of the first 454 instruments, but at much lower costs. The read number obtained from MiSeq runs will in many cases be sufficient to obtain a sequencing depth that allows to answer a research question. In a few studies, the Ion Torrent PGM was used to analyze bacterial or fungal communities based on reads with a length of approximately 100 or 200 bp (Whiteley et al., 2012; Kemler et al., 2013). Longer reads are meanwhile possible on this sequencer and a protocol for paired end sequencing is available (though not yet officially supported by the company), so that this platform can be an alternative to the previously mentioned systems for amplicon sequencing.

The taxonomic resolution that is achieved with reads from these sequencers is clearly lower compared to Sanger reads. Nearly full length 16S rRNA gene sequences were Gold standard for clone library analysis based on Sanger reads and have led to the comprehensive sequence databases we have today. They enable species differentiation and often even the distinction of different strains. In contrast, the short NGS reads provide a resolution at maximum down to genus level. It turned out that this is frequently sufficient, in particular if the method is used for comparative purposes and microbial communities in the samples of interest do not contain many closely related species. Compared to clone library analysis, DGGE or T-RFLP, NGS amplicon sequencing allows analysis at greater depth so that many more low-abundant taxa can be detected. Thus, despite the lower taxonomic resolution, sensitivity of the method is reached here due to sequencing depth. It is up to the researcher to decide which information, resolution of taxa or sequencing depth will be more important for a project.

In case taxon resolution is important, sequence information of longer reads is needed, and the Roche 454 sequencer is a better choice. With the latest software update to version 2.9, amplicon sequencing is supported under FLX+ run conditions. Under these conditions, 16S rRNA gene and ITS sequence reads with a mean length of 650 and 750 bp were obtained (Perazzolli et al., 2014). Even longer amplicons can be sequenced when using the PacBio RS platform. A recent study demonstrated the feasibility of amplicon sequencing for community analysis on this platform (Marshall et al., 2012; Fichot and Norman, 2013), although another study reported higher error rates for PacBio amplicon sequence reads compared to 454 reads of equal length, despite that fact that the CCS strategy was used (Mosher et al., 2013). Rather short movies of only 45 min were recorded in that study. By increasing the recording time higher quality sequences can be obtained. The current release of new sequencing chemistry and future improvements will enable the generation of higher quality sequences that will probably allow resolution even below genus level.

### Sequence read analysis of amplicon data

Diverse tools have been developed specifically for the analysis of amplicon data derived from metagenomic DNA, in particular for 454 data. This is largely due to the fact that many projects aim at an estimation of the microbial diversity within samples and along with this the indispensable need to differentiate between true diversity and sequencing errors (Sogin et al., 2006; Quince et al., 2009; Kunin et al., 2010). The fact that amplicon sequencing on NGS platforms is more and more widely applied has expedited the development of specific data analysis tools.

Based on the initial findings of Huse et al. (2007), who reported an accumulation of errors within a rather small subset of 454 reads, it became common to discard reads with one or more errors in the index and the target gene specific primer region. Likewise, reads with ambiguous basecalls (Ns), of unexpected length, with low quality scores or those that cannot be aligned to the gene of interest are assumed to be unspecific PCR products and are often removed (Huse et al., 2007, 2010; Kunin et al., 2010; Huse and Welch, 2011; Schloss et al., 2011; Zhou et al., 2011). Read trimming based on quality scores has also been applied to improve quality of 454 and Illumina data (Kunin et al., 2010; Caporaso et al., 2011; Schloss et al., 2011; Bokulich et al., 2013). In some studies singletons, i.e., sequence reads that occur only once, are removed from the datasets to further reduce the error rate (Caporaso et al., 2011; Shade et al., 2013).

Besides this quality filtering, specific algorithms are applied to improve quality. These aim at the correction of errors and the selection of representative sequence reads (=denoising), so that the number of reads or bases is not further decreased. The methods are based on the assumption that erroneous reads are representatives of more abundant error-free reads. Representative error free reads are identified and selected based on comparative sequence analysis, e.g., in the single-linkage preclustering (SLP) approach of Huse et al. (2010) or by the Pyrotagger tool (Kunin and Hugenholtz, 2010). Denoising algorithms such as PyroNoise, its successor AmpliconNoise or the DeNoiser analyze 454 flow grams (Quince et al., 2009; Reeder and Knight, 2010; Quince et al., 2011). The latter two algorithms have been reported to be very efficient, but demand much computational power, which has limited their application (Quince et al., 2011; Bragg et al., 2012). The SeqNoise algorithm, implemented in the software package Mothur, is less computationally demanding and therefore more often used. In comparative studies, the AmpliconNoise algorithm performed very well for OTU estimation (Quince et al., 2011; Bragg et al., 2012; Gaspar and Thomas, 2013). Critical analyses of different denoising tools demonstrated that parameters have to be chosen very carefully in order not to introduce bias by read modification during the generation of representative consensus reads. Default settings did not necessarily provide the best results (Bragg et al., 2012; Gaspar and Thomas, 2013).

The identification and elimination of chimeric sequences is another type of error that needs to be accounted for. Chimeric sequences originate during PCR and have been reported to contribute between 5 and 45% of a PCR product (Lahr and Katz, 2009; Haas et al., 2011). Available algorithms to eliminate these artifacts are Perseus, which was developed together with AmpliconNoise (Quince et al., 2011), ChimeraSlayer (Haas et al., 2011), or UCHIME (Edgar et al., 2011). While ChimeraSlayer needs a chimera-free reference database for chimera detection, Perseus is used without reference database. UCHIME offers both options and was reported to be faster compared to the other two methods (Edgar et al., 2011). UCHIME performed best in a comparative study when a reference database was used. Without reference database, UCHIME and Perseus performed equally well (Schloss et al., 2011). Considering that the use of database-independent methods is not limited by the quality and diversity of data in the reference database, database-free methods may be preferred.

Not all tools can be applied to Illumina datasets, for instance denoising algorithms that use 454 flow grams as input data. Moreover, some tools are computationally too demanding to be used for large Illumina datasets. A specific quality filtering approach for Illumina data was recently described using the “Quantitative Insights Into Microbial Ecology” (QIIME) toolkit (Bokulich et al., 2013). Other packages that combine the above mentioned analysis steps for error reduction with further analyses such as OTU clustering, taxonomy assignment or multiple sample comparison, are Mothur or the UPARSE pipeline (Caporaso et al., 2010; Schloss et al., 2011; Edgar, 2013).

## Application of NGS technologies in present studies of plant associated microorganisms

### Shotgun metagenomic studies

Until today, only a limited number of shotgun metagenomic studies of plant associated microorganisms exist (Table 3). Most of the studies are based on Roche 454 sequencing technology and generated a few hundred Mb of sequence data. In a very recent study of Mendes et al. (2014) the epiphytic rhizosphere microbiome of soybean was compared to that in bulk soil with regard to taxonomic and functional composition. A specific rhizosphere microbiota was observed, representing a subset of the taxonomic and functional diversity present in bulk soil. Moreover, functions that may be of benefit for the plant in terms of growth promotion and nutrition were detected, likewise as in a study of Sessitsch et al. (2012), who performed the first extensive metagenomic study of plant associated microorganisms, still using Sanger sequencing technology. In two other rhizosphere studies, the genomic basis for phosphorous acquisition was addressed. Unno and Shinano (2013) analyzed the rhizosphere metagenome of plants that showed enhanced growth in the presence of phytic acid and detected genes encoding enzymes related to phytic acid utilization such as alkaline phosphatase or citrate synthase. Chhabra et al. (2013) applied a targeted metagenomic approach by constructing a fosmid library in *Escherichia coli*, which was screened in an assay for mineral phosphate solubilization activity. Six positive clones were shotgun sequenced using 454 technology. Genes and operons with homology to phosphorous uptake systems, regulatory, and solubilization mechanisms were identified.

**Table 3:** **Metagenomic studies based on NGS technology that target the plant-associated microbiota**.

**Sequencing technology**	**Sequencing statistics**	**Plant compartment**	**Plant species and type of sample**	**Major findings**	**References**
Roche 454	3.2 million raw reads	Rhizosphere	Soybean (*Glycine max*) rhizosphere and bulk soil samples taken from mesocosm experiments with soil from soybean fields in Brazil	The rhizosphere community is selected from the bulk soil based on functions related to N, Fe, P, and K metabolism	Mendes et al., 2014
	2,472,359 filtered reads				
	Mean read number per sample 103,014				
	Mean read length 523 bp				
Roche 454	Not specified	Rhizosphere	Barley rhizosphere samples collected from an experimental field in Ireland with 15 years of barley monoculture under low-input mineral management regime	Identification of genes and operons involved in mineral phosphate solubilization in the rhizosphere	Chhabra et al., 2013
Illumina Miseq	15 million paired end reads	Phyllosphere	Samples from *Salmonella* enrichment cultures from outdoor grown tomato (*Solanum lycopersicum*) and tomato leaves and fruits	Differences in metagenomic composition of replicate phyllosphere enrichment cultures; enrichment of *Paenibacillus* on *Salmonella*-selective media	Ottesen et al., 2013a
	2.6 Gbp				
Roche 454	Not specified	Phyllosphere	Leaves, stems, roots, flowers, and fruits from outdoor grown tomato (*S. lycopersicum*)	Distinct microbial communities detected on different tomato plant organs	Ottesen et al., 2013b
		Rhizosphere			
Roche 454	8445 and 3799 filtered reads	Rhizosphere	Rhizosphere samples from greenhouse grown *Lotus japonicus*; plants of the same age but two different developmental stages grown in presence of phytic acid	Differences in microbial community composition in the rhizosphere of the differently developed plants; identification of genes related to phytic acid utilization	Unno and Shinano, 2013
	Mean read length 228 and 226 bp				
Roche 454	448 Mb sequence data	Phyllosphere	Leaf samples of tamarisk (*Tamarix nilotica*); datasets from soybean, (*G. max*), *Arabidopsis thaliana*, clover (*Trifolium repens*), and rice (*Oryza sativa*) included in analyses (Delmotte et al., 2009; Knief et al., 2012; Vorholt, 2012)	Diverse microbial rhodopsins detected in phyllosphere bacteria	Atamna-Ismaeel et al., 2012b
	Mean read length 357 bp			Detection of genes encoding proteins involved in anoxygenic photosynthesis (*bchY, pufM*, and *pufL*)	Atamna-Ismaeel et al., 2012a
Roche 454	832 and 396 Mb of sequence data per sample	Phyllosphere	Phyllosphere and rhizosphere sample of field grown rice (*O. sativa*), Philippines	Contrasting proteome patterns in phyllosphere and rhizosphere of rice	Knief et al., 2012
		Rhizosphere			
Roche 454	1,109,816 reads	Phyllosphere	Leaf samples from field grown soybean (*G. max*), Switzerland	High consistency in the microbial community composition and their proteomes on different host plants	Delmotte et al., 2009
	260 Mb of sequence data				
	235 bp mean read length				
Roche 454	419,571 reads	(Phyllosphere)	Psyllid infected with the endophyte “*Candidatus* Liberibacter asiaticus”	Complete genome sequence of the uncultured plant pathogen and insect symbiont “*Candidatus* Liberibacter asiaticus”	Duan et al., 2009
	216 bp mean read length				
	90,813,125 bp of sequence data				

Metagenomic data of phyllosphere associated microbial communities are available from soybean, rice, clover, *Arabidopsis thaliana, Tamarix*, and tomato (Delmotte et al., 2009; Atamna-Ismaeel et al., 2012a; Knief et al., 2012; Ottesen et al., 2013b). Some of these datasets were analyzed in combination with metaproteomic data obtained from the same sampling material (Delmotte et al., 2009; Knief et al., 2012). These analyses revealed high consistency in the metaproteomes of phyllosphere bacteria from different plant species. In agreement, microbial community composition as inferred from these phyllosphere metagenomic datasets revealed consistency in microbial community composition at phylum level (Vorholt, 2012). Comparative analyses of metagenomic and metaproteomic data of rice phyllosphere and rhizosphere samples revealed a higher complexity of the rhizosphere microbiota and a clearly distinct metagenomic and -proteomic composition (Knief et al., 2012). The phyllosphere metagenomic datasets generated in these studies were further used in combination with a metagenomic dataset from *Tamarix* associated phyllosphere bacteria to screen for photosynthetic genes that are known from other microorganisms to be involved in light-driven energy generation (Atamna-Ismaeel et al., 2012a,b).

Another kind of metagenomic project was performed with the aim to obtain a complete sequence of an unculturable plant pathogen, “*Candidatus* Liberibacter asiaticus,” which causes citrus huanglongbing (Duan et al., 2009). This pathogen is transmitted by phloem-feeding insects. Metagenomic DNA was extracted from a single Asian citrus psyllid and not from an infected plant, due to the fact that the natural enrichment of the target organism is higher in the insect. Extracted DNA was subjected to multiple displacement amplification prior to sequencing using 454 technology. Sequence read assembly resulted in 38 contigs for “*Candidatus* L. asiaticus,” which were identified by PCR confirmation reactions from a total of 1475 generated contigs. Gap closure was achieved by sequencing gap bridging PCR products. Genome analysis revealed a heavily reduced genome of this highly divergent member of the family Rhizobiacea, as it is seen frequently for microorganisms with a predominantly intracellular lifestyle.

### Amplicon sequencing studies

NGS technologies are increasingly often used for amplicon sequencing of bacterial and fungal marker genes in order to characterize the communities in the phyllosphere and rhizosphere. There are more than 100 rhizosphere and at least 37 phyllosphere articles published until now that have used these techniques (see Supplementary Material for a compilation of studies). The fast majority of these studies applied Roche 454 sequencing technology. Only few used the Ion PGM platform (Kavamura et al., 2013; Kemler et al., 2013; Yergeau et al., 2014) or the Illumina MiSeq (Jiang et al., 2013). A detailed look at the phyllosphere studies (Table S2) reveals that the generated read numbers in amplicon studies are mostly in a range from a few thousand to ten thousand reads per sample (Table S2). The obtained read length increased successively over time, along with the development of the Roche 454 sequencing platform. With the 454 FLX+ instrument a mean read length of 750 bp was recently obtained for 16S rRNA gene amplicons (Perazzolli et al., 2014).

NGS amplicon sequencing was so far almost exclusively applied for the analysis of bacterial or fungal communities. Bacterial phyllosphere communities were studied based on the 16S rRNA gene without a preference for one specific region within this gene (Table S2). Fungal communities were mostly analyzed based on the ITS region. The only functional marker gene that has been studied so far in plant associated microorganisms via amplicon sequencing is *chiA*, encoding a chitinase (Cretoiu et al., 2012). The aim of that particular study was an assessment of *chiA* gene diversity in different habitats, including rhizosphere samples from two arctic plant species. Analysis revealed that the rhizosphere of *Oxyria digyna* was among the samples with the highest *chiA* diversity.

Most amplicon sequencing studies in the phyllosphere were performed to describe and understand plant colonization by microorganisms. In particular biogeographic patterns, the role of the plant taxon for shaping communities and the temporal succession of the microbiota were addressed (e.g., Redford et al., 2010; Rastogi et al., 2012; Bokulich et al., 2014; Maignien et al., 2014). Also differences in the colonization of different plant compartments were analyzed (Bodenhausen et al., 2013; Ottesen et al., 2013b). The impact of specific treatments during plant cultivation such as irrigation were also addressed in some studies (Williams et al., 2013).

Amplicon sequencing projects performed in the rhizosphere addressed basically the same questions, i.e., aspects of biogeographical dispersal of rhizosphere microorganisms, or the impact of factors such as season, host plant species, soil type, or plant growth conditions (Gottel et al., 2011; Lundberg et al., 2012; Navarrete et al., 2013; Peiffer et al., 2013; Zhang et al., 2013). A major additional focus of rhizosphere studies is the analysis of endo- and ectomycorrhiza (Lumini et al., 2010; Dumbrell et al., 2011; Yu et al., 2012). It has become clear that the plant plays a significant role in shaping the associated microbiota and that root exudates are involved in this process (Badri et al., 2013), but to better understand how plants affect this process, plant mutant strains altered in root exudation or, in case of the phyllosphere with altered leaf surface properties, were analyzed (Badri et al., 2009; Reisberg et al., 2013). Furthermore, aspects of bioremediation, disease suppressiveness or possible impacts of herbicide application or of genetically modified plants have been addressed in rhizosphere studies (Barriuso et al., 2010; Rosenzweig et al., 2012; Dohrmann et al., 2013; Bell et al., 2014). All these exemplarily selected publications demonstrate the usefulness of NGS amplicon sequencing projects for studying microbial plant colonization. Future studies in this field will lead to an even better understanding of the factors that determine microbial plant colonization.

### Transcriptomic and metatranscriptomic studies

NGS technologies have not only stimulated research in the field of (meta-)genomics, but are also excellent tools to perform (meta-)transcriptomic analyses. The appearance of these technologies has boosted transcriptomic studies of plant associated microorganisms, until now in particular of pathogenic fungi (e.g., Tremblay et al., 2012; Weßling et al., 2012; Thakur et al., 2013). Both, Illumina and 454 technology have been used in such studies. NGS is of particular advantage when the organisms of interest have not been genome sequenced, which is a prerequisite for the alternative microarray analyses. In some studies, the transcriptome of the host and the pathogen were even analyzed in parallel (e.g., Fernandez et al., 2012; Zhuang et al., 2012). The success of such parallel analyses depends on the ratio of plant to fungal mRNA in the sequenced sample.

First metatranscriptomic studies of the whole plant associated microbial communities appeared just recently. Chaparro et al. (2014) analyzed the microbial metatranscriptome of the *Arabidopsis thaliana* rhizosphere at different plant development stages. They observed that microbial genes involved in metabolism of carbohydrates, amino acids and secondary metabolites changed over time in correspondence to root exudate patterns, which also changed over time. Yergeau et al. (2014) compared the microbial metatranscriptomic composition in the rhizosphere of willow with that in bulk soil in soils contaminated with organic pollutants. Different genes involved in hydrocarbon degradation were expressed in rhizosphere and bulk soil microbial communities. Genes related to carbon and amino-acid uptake and utilization were in general up-regulated in the rhizosphere.

Instead of an mRNA analysis, Turner et al. (2013) performed rRNA sequencing to characterize the active microbiota in the rhizosphere of different crops (wheat, oat, pea). Analyzing microbial communities based on rRNA instead of their rRNA genes is assumed to reflect the physiologically active microbiota in a sample and does not necessarily need extensive PCR amplification of the target molecules prior to library preparation, as demonstrated in that study. Clear differences were observed in the composition of the active prokaryotic and eukaryotic communities compared to bulk soil samples and between the different plant species. A strong response in the fungal community to plant produced anti-fungal avenacins was observed in the rhizosphere.

## Application of NGS technologies in future metagenomics studies will advance understanding in plant-microbe associations

With the availability of second generation sequencing platforms many of the limitations metagenomic studies had to deal with at the time when Sanger sequencing was the predominant technology have been overcome. In particular the preparation of metagenomic/sequencing libraries can be done much faster and the sequencing costs per base are drastically reduced. The new technologies allow much deeper sequencing of microbial communities, providing more information about identity and physiological potential of microbial communities associated to plants. Limitations of NGS approaches such as shorter reads and higher sequencing error rates can be largely compensated by using specifically designed sequence data analyses methods. Future developments of the sequencing technology will enable us to obtain even more and longer reads; the generation of sequence information will thus most likely not be a limiting factor in future studies, but enable to address the open questions in phyllosphere and rhizosphere research, as outlined in the introduction, in even more detail.

A current limitation of metagenomic sequencing studies is a high ratio of sequences that represent unknown genes of known or unknown organisms, and of sequences for which no homolog is found in public databases that would enable to infer further information. To improve the still challenging task of linking genes and thus function to phylogeny, genomic sequencing of representative pure cultures and the genetic and physiological characterization of strains will remain an important task. Genome sequencing projects of strain collections from the ecosystems of interest are one step further to overcome this limitation (Turnbaugh et al., 2007; Brown et al., 2012). Concerted sequencing of currently underrepresented organisms in databases, e.g., based on evolutionary relationship as in the GEBA project, will further improve databases (Wu et al., 2009). Likewise, advance in single cell genome sequencing has recently enabled the sequencing of yet uncultivated microorganisms; 200 bacterial and archaeal cells representing diverse largely uncharacterized phyla were successfully sequenced (Rinke et al., 2013). This genomic information will enable a more specific assignment of metagenome reads to taxa. (Meta-)transcriptomic and -proteomic studies based on known and well characterized representative model organisms under controlled conditions will contribute to a deeper understanding of microbial life in the phyllosphere.

The complementation of metagenomics data with metatranscriptomic, metaproteomic, and (meta-)metabolomic data will be one of the future goals to obtain a more complete view of the activities and the physiological potential of plant associated microbial communities under given conditions at systems level (Zhang et al., 2010; Knief et al., 2011; Segata et al., 2013). Such information is inevitable to build up models that can explain and predict microbially mediated processes and interactions in the phyllosphere and rhizosphere under different environmental conditions, including agricultural practices, responses to pathogen attack and disease, or to climate change.

### Conflict of interest statement

The author declares that the research was conducted in the absence of any commercial or financial relationships that could be construed as a potential conflict of interest.
