# Supplementary material for: Analysis of plant microbe interactions in the era of next generation sequencing technologies
Source: Front Plant Sci. 2014 May 21;5:216. doi: 10.3389/fpls.2014.00216 (PMC4033234; doi:10.3389/fpls.2014.00216)
Supplement: Supplementary file 1 [file DataSheet1.DOCX]

Appendix A

***Target Pseudo- Target Pseudo-***

***Words words words words***

Be-sen [broom] Bese Bo-den [ground] Bode

Bre-zel [pretzel] Breze Brüc-ke [bridge] Brücker

Dack-el [dachshund] Dacke Dau-men [thumb] Daume

Dok-tor [doctor] Dokte Do-se [can] Doser

Dra-che [dragon] Drachel Ga-bel [fork] Gaber

Gra-ben [ditch] Grabel Kä-fer [beetle] Käfe

Ket-te [chain] Kettel Kis-sen [cushion] Kisse

Kis-te [box] Kister Kof-fer [trunk] Koffe

Kü-che [kitchen] Kücher Kü-ken [chick] Küke

Mat-te [mat] Matter Mau-er [wall] Mauen

Mo-nat [month] Mone Mon-ster [monster] Monste

Mün-ze [coin] Münzig Na-del [needle] Nade

Na-gel [spike] Nagich Na-me [name] Namer

Nu-del [noodle] Nude Num-mer [number] Numme

Pei-tsche [whip] Peitschel Pin-sel [brush] Pinse

Po-ny [pony] Pone Pul-ver [powder] Pulve

Pup-pe [doll] Puppel Tan-te [aunt] Tanter

Ta-sche [bag] Tascher Tas-se [cup] Tasser

Tau-be [pigeon] Taubor Tel-ler [plate] Telle

Tep-pich [carpet] Teppe Ti-ger [tiger] Tige

Toch-ter [daughter] Tochte Tor-te [cake] Tortat

Trec-ker [tractor] Trecke Trep-pe [stairs] Trepper

Trom-mel [drum] Tromme

Appendix B

Significant interactions, including the factors *Phoneme Overlap (PO)*, *Stress* Overlap (SO) or both, are shown for each time window for the lateral electrodes.

| **Lateral**  **Electrodes** | 0 - 100 | 100 - 200 | 200 - 300 | 300 - 400 | 400 - 500 | 500 - 600 | 600 - 700 | 700 - 800 | 800 - 900 | 900 - 1000 |
| --- | --- | --- | --- | --- | --- | --- | --- | --- | --- | --- |
| **PO** |  | ****** | ******* |  |  | ***** |  |  |  |  |
| x Group |  |  |  |  |  | ***** | ****** | ***** |  |  |
| **Ph x H** |  |  | ****** |  |  |  |  | ***** |  |  |
| x Group |  |  |  |  |  |  |  |  |  |  |
| **Ph x R** |  | ***** | ****** | ******* |  | ****** | ******* | ******* | ******* | ****** |
| x Group |  |  |  | ***** |  |  |  | ***** |  |  |
| **Ph x H x R** |  |  |  |  | ***** |  | ***** | ****** | ***** |  |
| x Group |  |  |  |  |  |  |  |  | ***** |  |
| **SO** |  |  | ***** |  |  |  |  | ****** | ***** | ***** |
| x Group |  |  | ***** |  |  |  |  | ***** | ******* | ******* |
| **S x H** |  |  |  |  |  |  |  |  |  |  |
| x Group |  |  |  |  |  |  |  |  |  |  |
| **S x R** |  |  | ****** | ******* | ****** | ******* | ******* | ****** | ******* |  |
| x Group |  |  |  |  |  |  |  |  |  |  |
| **S x H x R** |  |  | ***** |  |  |  |  |  |  |  |
| x Group |  |  |  |  |  |  |  |  |  |  |
| **PO x SO** |  |  |  |  |  |  |  |  |  |  |
| x Group |  |  |  |  |  |  |  |  |  |  |
| **Ph x S x H** |  |  |  |  |  |  |  |  |  |  |
| x Group |  |  |  |  |  |  |  |  |  |  |
| **Ph x S x R** |  |  |  | ***** |  |  |  |  |  |  |
| x Group |  |  |  |  |  |  |  |  |  |  |
| **Ph x S x H x R** |  |  |  |  |  |  |  |  |  |  |

****p* < .001, ***p* < .01; **p* < .05
